# Supplementary material for: A mechanism of global gene expression regulation is disrupted by multiple disease states and drug treatments
Source: PLoS One. 2025 May 8;20(5):e0317071. doi: 10.1371/journal.pone.0317071 (PMC12061403; doi:10.1371/journal.pone.0317071)
Supplement: S1 Table — The associated publications are referenced and information on the breakdown of sample numbers in case:control studies provided. (DOCX) [file pone.0317071.s001.docx]

| GEO accession | Description | Notes | Reference |
| --- | --- | --- | --- |
| **Human disease studies** |  |  |  |
| GSE29429 | HIV infection. (Healthy uninfected, N=17; infected with low viral load, N=12; infected with high viral load N=18,) | Blood. Viral load > 100,000 considered ‘high’. | (Blazkova et al., 2016) |
| GSE20948 | HepC infection. (Huh7 cells infected with JFH-1 strain or mock infected, N=3 each) | Cells. 12-hour response time plotted | (Blackham et al., 2010) |
| GSE45924 | EBV infection (acute infection, N=3; latent infection, N=12; seronegative, N=9). | Blood | (Dunmire et al., 2014) |
| GSE63060 | Alzheimer’s Disease/Mild Cognitive Impairment.  (Healthy controls, N=134; AD, N=139; MCI, N=109) | Blood | (Sood et al., 2015) |
| GSE63061 | Alzheimer’s Disease/Mild Cognitive Impairment.  (Healthy controls, N=102; AD, N=145; MCI, N=80) | Blood | (Sood et al., 2015) |
| GSE38484 | Schizophrenia. (Healthy controls, N=96; Schizophrenia, N=106) | Blood | (de Jong et al., 2012) |
| GSE93272 | Rheumatoid Arthritis. (Healthy controls, N=43; RA, N=232 or 89 when age-matched) | Blood | (Tasaki et al., 2018) |
| GSE41890 | Multiple Sclerosis. (Healthy controls, N=24; MS in relapse, N=22; MS in remission, N=22) | Blood | (Irizar et al., 2014) |
| GSE46449 | Bipolar Disorder. (Healthy controls, N=24; Bipolar Disorder, N=30) | Blood | (Clelland et al., 2013) |
| GSE99039 | Parkinson’s Disease. (Healthy controls, N=233; PD idiopathic, N=205; PD genetic, N=41) | Blood | (Shamir et al., 2017) |
| GSE169568 | Inflammatory bowel conditions. (Healthy controls, N=30 (15M/15F); SC, N=65 (M26/F39); UC, N=58 (M31/F27) | Blood | (Juzenas et al., 2022) |
| GSE42133 | Autism Spectrum Disorder. (Healthy controls, N=56; ASD, N=91) | Blood | (Pramparo et al., 2015) |
| GSE111175 | Autism Spectrum Disorder. (Healthy controls, N=70; ASD, N=28) | Blood | (Gazestani et al., 2019) |
| GSE25507 | Autism Spectrum Disorder. (Healthy controls, N=64; ASD, N=82) | Blood | (Alter et al., 2011) |
| GSE26927 | Parkinson’s Disease and Amyotrophic Lateral Sclerosis/Motor Neuron Disease. (Healthy controls, N=8; PD, N=12)  (Healthy controls, N=10; Amyotrophic Lateral Sclerosis/Motor Neuron Disease, N=10) | Substantia nigra, brain (PD)  Spinal cord (ALS) | (Durrenberger et al., 2015) |
| GSE3307 | Neuromuscular conditions. Normal human skeletal muscle, N=13; Amyotrophic Lateral Sclerosis/Motor Neuron Disease, N=9; Acute Quadriplegic Myopathy, N=3; Becker Muscular Dystrophy, N=5; Limb Girdle Muscular Dystrophy, N=22. | Skeletal muscle. (No significance for DMD, JDM, and FSHD) | (Bakay et al., 2006) |
| GSE36980 | Alzheimer’s disease. Frontal cortex (Healthy control, N=18; AD, 15). Temporal cortex (Healthy controls, N=19; AD, N=10). Hippocampus (Healthy controls, N=10; AD, N=8) | Brain tissue | (Hokama et al., 2014) |
| GSE118553 | Alzheimer’s disease. Frontal cortex (Healthy control, N=18; Asymp. AD, N=33; AD, 15). Temporal cortex (Healthy controls, N=31; Asymp. AD, N=32; AD, N=52). Cerebellum (Healthy controls, N=22; Asymp. AD, N=32; AD, N=38). Entorhinal Cortex (Healthy controls, N=24; Asymp. AD, N=37; AD, N=37). | Brain tissue | (Patel et al., 2019) |
| GSE24345 | Lesch-Nyhan Syndrome (Control, N=3; Knock-down, N=3) | Fibroblast knock-down of *HPRT* gene | (Kang et al., 2011) |
| GSE13732 | Clinically Isolated Syndrome. (Healthy controls, N=30; CIS, N=39).[baseline values used] | CD4+ T-cells | (Corvol et al., 2008) |
| GSE48113 | Circadian day. (Healthy controls, N=22). Analysed as three pooled blood samples from the volunteers: 61 samples at night, 49 in afternoon/evening, 44 in morning. | Blood | (Archer et al., 2014) |
| GSE234368 | Menstrual cycle. (stage 1 [menstruation], N=12; stage 2, N=5; stage 3, N=81; stage 4 [ovulation], N=20; stage 5, N=51; stage 6, N=61; stage 7, N=33). | Endometrial cells | (Teh et al., 2023) |
| GSE132903 | Alzheimer’s disease. Middle temporal gyrus (BA21). (Healthy controls, N=98; AD, N=97). | Brain tissue | (Piras et al., 2019) |
| GSE5281 | Alzheimer’s disease. Entorhinal cortex: (Healthy controls, N=12 with one removed from dataset for being ~8 sd from mean; AD, N=10). Hippocampus (Healthy controls, N=13; AD, N=10). Middle temporal gyrus (Healthy controls, N=12; AD, N=16). Posterior cingulate (Healthy controls, N=13; AD, N=9). Superior frontal gyrus (Healthy controls, N=11; AD, N=23). Primary visual cortex (Healthy controls, N=12; AD, N=19). | Brain tissue | (Liang et al., 2007) |
| GSE73129 | Schizophrenia. (Healthy controls, N=19; SCZ, N=19). | Olfactory neuroepithelium | (Horiuchi et al., 2016) |
| GSE5392 | Bipolar disorder. Dorsolateral prefrontal cortex (Healthy controls, N=31; BPD, N=30). Orbitofrontal cortex (Healthy controls, N=11; BPD, N=10). | Brain tissue | (Ryan et al., 2006) |
| GSE12649 | Bipolar disorder and schizophrenia. Prefrontal cortex (Healthy controls, N=34; BPD, N=33; SCZ, N=35). | Brain tissue | (Iwamoto et al., 2005) |
| GSE38322 | Autism spectrum disorder. Cerebellum (Healthy controls, N=8; ASD, N=8). Occipital cortex (Healthy controls, N=6; ASD, N=4). | Brain tissue | (Ginsberg et al., 2012) |
| GSE75303 | Rett syndrome. Frontal cortex (Healthy controls, N=3; RTT, N=3). Temporal cortex (Healthy controls, N=3; RTT, N=3). Brain samples pooled 6 v 6 for statistical analysis | Brain tissue | (Lin et al., 2016) |
| GSE131281 | Multiple sclerosis. Frontal cortex (Healthy controls, N=21; MS, N=28). Parietal cortex (Healthy controls, N=21; MS, N=36). | Brain tissue | (Enz et al., 2020) |
|  |  |  |  |
| **Mouse tissues** |  |  |  |
| GSE24207 |  |  | (Thorrez et al., 2011) |
| GSE10246 |  |  | (Lattin et al., 2008) |
| GSE9954 |  |  | (Thorrez et al., 2008) |
|  |  |  |  |
| **Drug studies** |  |  |  |
| GSE59923/ GSE59913/ GSE57815/etc | DrugMatrix toxicology data | Data available from:  https://ntp.niehs.nih.gov/data/drugmatrix | (Cong et al., 2024) |
| GSE119291 | iPS neural progenitor cells | Luminex | (Readhead et al., 2018) |
| GSE187001 | iPS cardiomyogenic |  | (Cherianidou et al., 2022) |
| GSE51952 | HepG2 cells |  | (Van den Hof et al., 2014) |
| GSE44783 | CD-1 mouse liver | Male and female mice. Males seem more susceptible to drug effects. | (Eichner et al., 2013) |

ALTER, M. D., KHARKAR, R., RAMSEY, K. E., CRAIG, D. W., MELMED, R. D., GREBE, T. A., BAY, R. C., OBER-REYNOLDS, S., KIRWAN, J., JONES, J. J., TURNER, J. B., HEN, R. & STEPHAN, D. A. 2011. Autism and increased paternal age related changes in global levels of gene expression regulation. *PLoS One,* 6**,** e16715.

ARCHER, S. N., LAING, E. E., MOLLER-LEVET, C. S., VAN DER VEEN, D. R., BUCCA, G., LAZAR, A. S., SANTHI, N., SLAK, A., KABILJO, R., VON SCHANTZ, M., SMITH, C. P. & DIJK, D. J. 2014. Mistimed sleep disrupts circadian regulation of the human transcriptome. *Proc Natl Acad Sci U S A,* 111**,** E682-91.

BAKAY, M., WANG, Z., MELCON, G., SCHILTZ, L., XUAN, J., ZHAO, P., SARTORELLI, V., SEO, J., PEGORARO, E., ANGELINI, C., SHNEIDERMAN, B., ESCOLAR, D., CHEN, Y. W., WINOKUR, S. T., PACHMAN, L. M., FAN, C., MANDLER, R., NEVO, Y., GORDON, E., ZHU, Y., DONG, Y., WANG, Y. & HOFFMAN, E. P. 2006. Nuclear envelope dystrophies show a transcriptional fingerprint suggesting disruption of Rb-MyoD pathways in muscle regeneration. *Brain,* 129**,** 996-1013.

BLACKHAM, S., BAILLIE, A., AL-HABABI, F., REMLINGER, K., YOU, S., HAMATAKE, R. & MCGARVEY, M. J. 2010. Gene expression profiling indicates the roles of host oxidative stress, apoptosis, lipid metabolism, and intracellular transport genes in the replication of hepatitis C virus. *J Virol,* 84**,** 5404-14.

BLAZKOVA, J., BOUGHORBEL, S., PRESNELL, S., QUINN, C. & CHAUSSABEL, D. 2016. A curated transcriptome dataset collection to investigate the immunobiology of HIV infection. *F1000Res,* 5**,** 327.

CHERIANIDOU, A., SEIDEL, F., KAPPENBERG, F., DRESER, N., BLUM, J., WALDMANN, T., BLUTHGEN, N., MEISIG, J., MADJAR, K., HENRY, M., ROTSHTEYN, T., MARCHAN, R., EDLUND, K., LEIST, M., RAHNENFUHRER, J., SACHINIDIS, A. & HENGSTLER, J. G. 2022. Classification of Developmental Toxicants in a Human iPSC Transcriptomics-Based Test. *Chem Res Toxicol,* 35**,** 760-773.

CLELLAND, C. L., READ, L. L., PANEK, L. J., NADRICH, R. H., BANCROFT, C. & CLELLAND, J. D. 2013. Utilization of never-medicated bipolar disorder patients towards development and validation of a peripheral biomarker profile. *PLoS One,* 8**,** e69082.

CONG, G., PATTON, R. M., CHAO, F., SVOBODA, D. L., CASEY, W. M., SCHMITT, C. P., MURPHY, C., ERICKSON, J. N., COMBS, P. & AUERBACH, S. S. 2024. Completion of the DrugMatrix Toxicogenomics Database using ToxCompl. *bioRxiv***,** 2024.03.26.586669.

CORVOL, J. C., PELLETIER, D., HENRY, R. G., CAILLIER, S. J., WANG, J., PAPPAS, D., CASAZZA, S., OKUDA, D. T., HAUSER, S. L., OKSENBERG, J. R. & BARANZINI, S. E. 2008. Abrogation of T cell quiescence characterizes patients at high risk for multiple sclerosis after the initial neurological event. *Proc Natl Acad Sci U S A,* 105**,** 11839-44.

DE JONG, S., BOKS, M. P., FULLER, T. F., STRENGMAN, E., JANSON, E., DE KOVEL, C. G., ORI, A. P., VI, N., MULDER, F., BLOM, J. D., GLENTHOJ, B., SCHUBART, C. D., CAHN, W., KAHN, R. S., HORVATH, S. & OPHOFF, R. A. 2012. A gene co-expression network in whole blood of schizophrenia patients is independent of antipsychotic-use and enriched for brain-expressed genes. *PLoS One,* 7**,** e39498.

DUNMIRE, S. K., ODUMADE, O. A., PORTER, J. L., REYES-GENERE, J., SCHMELING, D. O., BILGIC, H., FAN, D., BAECHLER, E. C., BALFOUR, H. H., JR. & HOGQUIST, K. A. 2014. Primary EBV infection induces an expression profile distinct from other viruses but similar to hemophagocytic syndromes. *PLoS One,* 9**,** e85422.

DURRENBERGER, P. F., FERNANDO, F. S., KASHEFI, S. N., BONNERT, T. P., SEILHEAN, D., NAIT-OUMESMAR, B., SCHMITT, A., GEBICKE-HAERTER, P. J., FALKAI, P., GRUNBLATT, E., PALKOVITS, M., ARZBERGER, T., KRETZSCHMAR, H., DEXTER, D. T. & REYNOLDS, R. 2015. Common mechanisms in neurodegeneration and neuroinflammation: a BrainNet Europe gene expression microarray study. *J Neural Transm (Vienna),* 122**,** 1055-68.

EICHNER, J., KOSSLER, N., WRZODEK, C., KALKUHL, A., BACH TOFT, D., OSTENFELDT, N., RICHARD, V. & ZELL, A. 2013. A toxicogenomic approach for the prediction of murine hepatocarcinogenesis using ensemble feature selection. *PLoS One,* 8**,** e73938.

ENZ, L. S., ZEIS, T., SCHMID, D., GEIER, F., VAN DER MEER, F., STEINER, G., CERTA, U., BINDER, T. M. C., STADELMANN, C., MARTIN, R. & SCHAEREN-WIEMERS, N. 2020. Increased HLA-DR expression and cortical demyelination in MS links with HLA-DR15. *Neurol Neuroimmunol Neuroinflamm,* 7.

GAZESTANI, V. H., PRAMPARO, T., NALABOLU, S., KELLMAN, B. P., MURRAY, S., LOPEZ, L., PIERCE, K., COURCHESNE, E. & LEWIS, N. E. 2019. A perturbed gene network containing PI3K-AKT, RAS-ERK and WNT-beta-catenin pathways in leukocytes is linked to ASD genetics and symptom severity. *Nat Neurosci,* 22**,** 1624-1634.

GINSBERG, M. R., RUBIN, R. A., FALCONE, T., TING, A. H. & NATOWICZ, M. R. 2012. Brain transcriptional and epigenetic associations with autism. *PLoS One,* 7**,** e44736.

HOKAMA, M., OKA, S., LEON, J., NINOMIYA, T., HONDA, H., SASAKI, K., IWAKI, T., OHARA, T., SASAKI, T., LAFERLA, F. M., KIYOHARA, Y. & NAKABEPPU, Y. 2014. Altered expression of diabetes-related genes in Alzheimer's disease brains: the Hisayama study. *Cereb Cortex,* 24**,** 2476-88.

HORIUCHI, Y., KONDO, M. A., OKADA, K., TAKAYANAGI, Y., TANAKA, T., HO, T., VARVARIS, M., TAJINDA, K., HIYAMA, H., NI, K., COLANTUONI, C., SCHRETLEN, D., CASCELLA, N. G., PEVSNER, J., ISHIZUKA, K. & SAWA, A. 2016. Molecular signatures associated with cognitive deficits in schizophrenia: a study of biopsied olfactory neural epithelium. *Transl Psychiatry,* 6**,** e915.

IRIZAR, H., MUNOZ-CULLA, M., SEPULVEDA, L., SAENZ-CUESTA, M., PRADA, A., CASTILLO-TRIVINO, T., ZAMORA-LOPEZ, G., LOPEZ DE MUNAIN, A., OLASCOAGA, J. & OTAEGUI, D. 2014. Transcriptomic profile reveals gender-specific molecular mechanisms driving multiple sclerosis progression. *PLoS One,* 9**,** e90482.

IWAMOTO, K., BUNDO, M. & KATO, T. 2005. Altered expression of mitochondria-related genes in postmortem brains of patients with bipolar disorder or schizophrenia, as revealed by large-scale DNA microarray analysis. *Hum Mol Genet,* 14**,** 241-53.

JUZENAS, S., HUBENTHAL, M., LINDQVIST, C. M., KRUSE, R., STEIERT, T. A., DEGENHARDT, F., SCHULTE, D., NIKOLAUS, S., ZEISSIG, S., BERGEMALM, D., ALMER, S., HJORTSWANG, H., BRESSO, F., GROUP, S. I. W., STRUNING, N., KUPCINSKAS, J., KELLER, A., LIEB, W., ROSENSTIEL, P., SCHREIBER, S., D'AMATO, M., HALFVARSON, J., HEMMRICH-STANISAK, G. & FRANKE, A. 2022. Detailed Transcriptional Landscape of Peripheral Blood Points to Increased Neutrophil Activation in Treatment-Naive Inflammatory Bowel Disease. *J Crohns Colitis,* 16**,** 1097-1109.

KANG, T. H., GUIBINGA, G. H., JINNAH, H. A. & FRIEDMANN, T. 2011. HPRT deficiency coordinately dysregulates canonical Wnt and presenilin-1 signaling: a neuro-developmental regulatory role for a housekeeping gene? *PLoS One,* 6**,** e16572.

LATTIN, J. E., SCHRODER, K., SU, A. I., WALKER, J. R., ZHANG, J., WILTSHIRE, T., SAIJO, K., GLASS, C. K., HUME, D. A., KELLIE, S. & SWEET, M. J. 2008. Expression analysis of G Protein-Coupled Receptors in mouse macrophages. *Immunome Res,* 4**,** 5.

LIANG, W. S., DUNCKLEY, T., BEACH, T. G., GROVER, A., MASTROENI, D., WALKER, D. G., CASELLI, R. J., KUKULL, W. A., MCKEEL, D., MORRIS, J. C., HULETTE, C., SCHMECHEL, D., ALEXANDER, G. E., REIMAN, E. M., ROGERS, J. & STEPHAN, D. A. 2007. Gene expression profiles in anatomically and functionally distinct regions of the normal aged human brain. *Physiol Genomics,* 28**,** 311-22.

LIN, P., NICHOLLS, L., ASSAREH, H., FANG, Z., AMOS, T. G., EDWARDS, R. J., ASSAREH, A. A. & VOINEAGU, I. 2016. Transcriptome analysis of human brain tissue identifies reduced expression of complement complex C1Q Genes in Rett syndrome. *BMC Genomics,* 17**,** 427.

PATEL, H., HODGES, A. K., CURTIS, C., LEE, S. H., TROAKES, C., DOBSON, R. J. B. & NEWHOUSE, S. J. 2019. Transcriptomic analysis of probable asymptomatic and symptomatic alzheimer brains. *Brain Behav Immun,* 80**,** 644-656.

PIRAS, I. S., KRATE, J., DELVAUX, E., NOLZ, J., MASTROENI, D. F., PERSICO, A. M., JEPSEN, W. M., BEACH, T. G., HUENTELMAN, M. J. & COLEMAN, P. D. 2019. Transcriptome Changes in the Alzheimer's Disease Middle Temporal Gyrus: Importance of RNA Metabolism and Mitochondria-Associated Membrane Genes. *J Alzheimers Dis,* 70**,** 691-713.

PRAMPARO, T., PIERCE, K., LOMBARDO, M. V., CARTER BARNES, C., MARINERO, S., AHRENS-BARBEAU, C., MURRAY, S. S., LOPEZ, L., XU, R. & COURCHESNE, E. 2015. Prediction of autism by translation and immune/inflammation coexpressed genes in toddlers from pediatric community practices. *JAMA Psychiatry,* 72**,** 386-94.

READHEAD, B., HARTLEY, B. J., EASTWOOD, B. J., COLLIER, D. A., EVANS, D., FARIAS, R., HE, C., HOFFMAN, G., SKLAR, P., DUDLEY, J. T., SCHADT, E. E., SAVIC, R. & BRENNAND, K. J. 2018. Expression-based drug screening of neural progenitor cells from individuals with schizophrenia. *Nat Commun,* 9**,** 4412.

RYAN, M. M., LOCKSTONE, H. E., HUFFAKER, S. J., WAYLAND, M. T., WEBSTER, M. J. & BAHN, S. 2006. Gene expression analysis of bipolar disorder reveals downregulation of the ubiquitin cycle and alterations in synaptic genes. *Mol Psychiatry,* 11**,** 965-78.

SHAMIR, R., KLEIN, C., AMAR, D., VOLLSTEDT, E. J., BONIN, M., USENOVIC, M., WONG, Y. C., MAVER, A., POTHS, S., SAFER, H., CORVOL, J. C., LESAGE, S., LAVI, O., DEUSCHL, G., KUHLENBAEUMER, G., PAWLACK, H., ULITSKY, I., KASTEN, M., RIESS, O., BRICE, A., PETERLIN, B. & KRAINC, D. 2017. Analysis of blood-based gene expression in idiopathic Parkinson disease. *Neurology,* 89**,** 1676-1683.

SOOD, S., GALLAGHER, I. J., LUNNON, K., RULLMAN, E., KEOHANE, A., CROSSLAND, H., PHILLIPS, B. E., CEDERHOLM, T., JENSEN, T., VAN LOON, L. J., LANNFELT, L., KRAUS, W. E., ATHERTON, P. J., HOWARD, R., GUSTAFSSON, T., HODGES, A. & TIMMONS, J. A. 2015. A novel multi-tissue RNA diagnostic of healthy ageing relates to cognitive health status. *Genome Biol,* 16**,** 185.

TASAKI, S., SUZUKI, K., KASSAI, Y., TAKESHITA, M., MUROTA, A., KONDO, Y., ANDO, T., NAKAYAMA, Y., OKUZONO, Y., TAKIGUCHI, M., KURISU, R., MIYAZAKI, T., YOSHIMOTO, K., YASUOKA, H., YAMAOKA, K., MORITA, R., YOSHIMURA, A., TOYOSHIBA, H. & TAKEUCHI, T. 2018. Multi-omics monitoring of drug response in rheumatoid arthritis in pursuit of molecular remission. *Nat Commun,* 9**,** 2755.

TEH, W. T., CHUNG, J., HOLDSWORTH-CARSON, S. J., DONOGHUE, J. F., HEALEY, M., REES, H. C., BITTINGER, S., OBERS, V., SLOGGETT, C., KENDARSARI, R., FUNG, J. N., MORTLOCK, S., MONTGOMERY, G. W., GIRLING, J. E. & ROGERS, P. A. W. 2023. A molecular staging model for accurately dating the endometrial biopsy. *Nat Commun,* 14**,** 6222.

THORREZ, L., LAUDADIO, I., VAN DEUN, K., QUINTENS, R., HENDRICKX, N., GRANVIK, M., LEMAIRE, K., SCHRAENEN, A., VAN LOMMEL, L., LEHNERT, S., AGUAYO-MAZZUCATO, C., CHENG-XUE, R., GILON, P., VAN MECHELEN, I., BONNER-WEIR, S., LEMAIGRE, F. & SCHUIT, F. 2011. Tissue-specific disallowance of housekeeping genes: the other face of cell differentiation. *Genome Res,* 21**,** 95-105.

THORREZ, L., VAN DEUN, K., TRANCHEVENT, L. C., VAN LOMMEL, L., ENGELEN, K., MARCHAL, K., MOREAU, Y., VAN MECHELEN, I. & SCHUIT, F. 2008. Using ribosomal protein genes as reference: a tale of caution. *PLoS One,* 3**,** e1854.

VAN DEN HOF, W. F., COONEN, M. L., VAN HERWIJNEN, M., BRAUERS, K., WODZIG, W. K., VAN DELFT, J. H. & KLEINJANS, J. C. 2014. Classification of hepatotoxicants using HepG2 cells: A proof of principle study. *Chem Res Toxicol,* 27**,** 433-42.
