# Supplementary figures and images for: A mechanism of global gene expression regulation is disrupted by multiple disease states and drug treatments
Source: PLoS One. 2025 May 8;20(5):e0317071. doi: 10.1371/journal.pone.0317071 (PMC12061403; doi:10.1371/journal.pone.0317071)

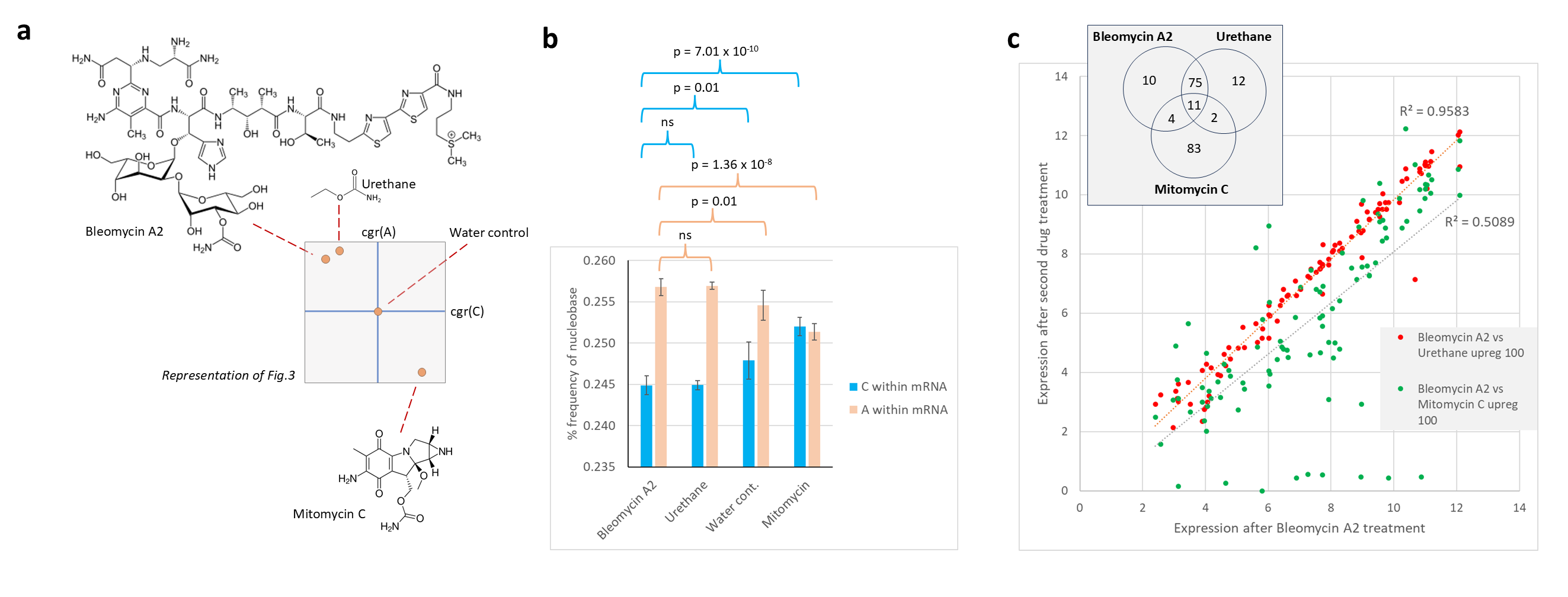

Supplement: S1 Figure — In (a) a reconstruction of Fig 3 is shown to draw attention to the relative global expression profile effect changes resulting from treatment with three drugs, bleomycin A2, urethane, and mitomycin C. The chemical structures of these three drugs are shown to highlight their different direct pharmacological actions. In (b) the relative proportions of two of the four nucleobases (C and A) have been estimated within the total expressed mRNA population (assumed to be in equilibrium with free pools of cellular nucleobases) by multiplying each transcript’s expression level by its base composition frequency and then summing all products. For each drug treatment, the specific nucleobase investigated was expressed as a proportion of the total of all four sums. Histogram values are the averages, and the error bars are the 95% confidence intervals, of nucleobase proportions after replicated drug treatments. T-tests were used to determine if nucleobase proportion averages (C – blue, A – orange) were significantly different between treatments or the water controls. In (c) the identities of the top 100 upregulated transcripts for each of the three drug treatments were compared for overlaps and the results visualised in the Venn diagram inset. The expression levels of the top 100 most upregulated bleomycin A2 transcripts were compared between bleomycin A2 and urethane (red), and bleomycin A2 and mitomycin c (green). Linear trendlines and R2 values are shown for both correlations. (TIF) [file pone.0317071.s003.tif]

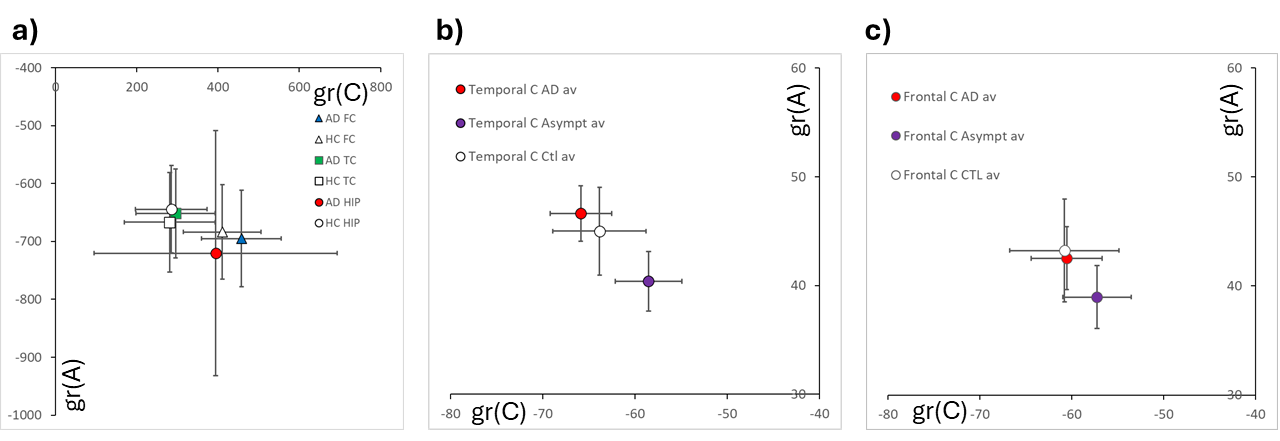

Supplement: S2 Figure — Study GSE36980 (a) comprised 15 frontal cortex samples from individuals diagnosed with Alzheimer’s disease (AD FC), 18 frontal cortex samples from healthy controls (HC FC), 10 temporal cortex samples from individuals diagnosed with Alzheimer’s disease (AD TC), 19 temporal cortex samples from healthy controls (HC TC), 8 hippocampal samples from individuals diagnosed with Alzheimer’s disease (AD HIP), and 10 hippocampal samples from healthy controls (HC HIP). Study GSE118553 (b,c) comprised 52 temporal cortex samples from individuals diagnosed with Alzheimer’s disease (Temporal C AD av), 32 temporal cortex samples from individuals who were asymptomatic (intact cognition but pathology consistent with AD, Temporal C Asympt av), 31 temporal cortex samples from healthy control individuals (Temporal C Ctl av), 40 frontal cortex samples from individuals diagnosed with Alzheimer’s disease (Frontal C AD av), 33 frontal cortex samples from individuals who were asymptomatic (Frontal C Asympt av), and 23 frontal cortex samples from healthy control individuals (Frontal C Ctl av).Symbols represent average gr(C) and gr(A) values for a tissue/diagnosis and are plotted on the same type of scatterplots as Figure 5, with error bars corresponding to 95% confidence intervals. (TIFF) [file pone.0317071.s004.tiff]
