# Supplementary material for: A mechanism of global gene expression regulation is disrupted by multiple disease states and drug treatments
Source: PLoS One. 2025 May 8;20(5):e0317071. doi: 10.1371/journal.pone.0317071 (PMC12061403; doi:10.1371/journal.pone.0317071)
Supplement: Attachment — Submitted filename: PONE-D-24-58748 - Reviewer Report.docx [file pone.0317071.s005.docx]

**PLOS ONE Review – PONE-D-24-58748**

**Pickard – Gene expression is globally regulated by interacting nucleobase supply and mRNA composition demand: a mechanism disrupted by multiple disease states and drug treatments**

Thank you for the opportunity to review this paper, which reports on the potential role of nucleobase supply in determining mRNA expression levels. This is a reasonable proposition, as potential restrictions in this supply would reasonably be expected to force the cell to prioritise the mRNA complement, and by extension, the protein profile of the cell. The strength of this paper lies in the amount of work that has been done to demonstrate a correlation between the types of mRNA transcripts across cell types and the prevalence of certain bases within those transcripts. Unfortunately, in places, the text reads as if the data is being constrained to justify the hypothesis, rather than the conclusions arising directly from the data. Additionally, some of the conclusions seem to stretch a bit too far, particularly considering the absence of direct measures of nucleobase availability and promotor activity. There are other aspects of this piece of scholarship that would benefit from clarification, additional detail, and/or revision. Some specific comments are provided below.

**Abstract**

- what is meant by *“…simple numerical outputs”*? In the context of an Abstract, this comes across as a bit vague and uninformative

**Introduction**

- the Abstract makes specific mention of the use of Lesch-Nyhan syndrome as a model system, but nothing has been provided in the Introduction to address this condition and how it is relevant to this study. Since the authors have emphasised this condition in the Abstract, it would seem important background for the ability to evaluate the findings presented.
  - However, considering the data from the *HPRT* knockdown cells doesn’t appear until Figure 7 and that there is only a single paragraph about Lesch-Nyhan syndrome and the implications for this study, it seems odd that this would be highlighted in the Abstract. It is suggested that the author reconsider highlighting this condition here.
- the author posits the question of whether reduced availability of nucleobases, independent of promoter activity, change gene expression levels (lines 59-60), but then uses the phrasing *“nucleobase composition”* (line 72), which is addressed in the Results as the frequency of individual bases within the mRNA transcripts, not the availability of these bases – clarification would be beneficial

**Methods**

- since there aren’t any direct measures of free nucleobase quantities or promotor activity in this section, it would be helpful for the author to explain how the frequency of individual bases within the transcripts is transformed to demonstrate unequivocally that the promoters do not contribute to altered mRNA levels
- were the bins independent of the tissue type is the early studies?
- there appears to be a contradiction between the information in the Methods section, lines 716-717, which states that transcripts, *“…were divided into 25 equal-sized bins from the lowest to highest frequency…”* and the comment in line 86 that there were *“…25 equal-sized bins each containing hundreds of* ***randomly allocated transcripts****”* [emphasis mine]

**Results**

- repeatedly across Figures 1-3, the author draws conclusions about the nucleobase supply, correlating it with nucleobase composition in the absence of evidence that the supply is perturbed. I have chosen not to enumerate all of the statements of concern, as this would have run to many pages, but certain key issues are identified below.
  - it would be beneficial for the author to restrict themselves to drugs that are known to alter the availability of various nucleobases and ascertain from transcriptomes isolated from control and treated cells whether there are substantive changes in the types of transcripts and their quantities
    - Figure 4, which should provide evidence in this regard, is incredibly difficult to read but fluorouracil, for example, had an asymmetrical effect, the meaning of which is left unexplained, and would be expected to skew the transcript profile
      - also, what constitutes an “outlier” is unclear
  - drugs that are alkylating agents will change the availability of certain transcripts due to the DNA damage caused, but how does this correlate with the availability of nucleobases themselves?
  - it is recommended that the author reconsider the order in which the data is presented, as this would strengthen the correlations being drawn between the data from the *HPRT* knockdown cells and certain drugs with the findings in Figures 1-3
- it is unclear how coding DNA sequences that result in proteins rich in essential amino acids provides any insight into nucleobase availability (lines183-187) or why this study was needed to illustrate a skew in the nucleobases required for the different amino acids. Simply counting the different bases in the genetic code across the types of amino acids would have demonstrated this without the need for experimentation and therefore, the comment that it was “*unexpected”* seems unusual. I have obviously missed the point and therefore, would find it helpful for the author to provide additional information to explain their meaning.
- untranslated regions are known to regulate expression (e.g. 5’UTR regulates ribosome recruitment, translation efficiency, *etc*.) and therefore, protein availability, so how does the nucleobase *composition* of 5’UTR or 3’UTR provide insights into nucleobase *availability* (lines 202-205)?
- limitations of this work (lines 221-223) belong in the Discussion, not the Results
- regarding the number of replicate treatments (line 241), what does *“approximately twelve replicate treatments”* mean? Was there a range of 3-21 resulting in an average of 12, or was there a range of 11-12?
  - Additionally, by *“replicates”*, does the author mean replicates within a single experiment or is *“replicates”* being used in place of the number of experiments (i.e. *“n”*)?
- the strongest data in this study comes from the HPRT knockdown cells (lines 373-377). That said, correlation does not equal causation, and therefore, the fact that the C+A- global transcriptome change seen in these cells resembles that seen in other data does not mean that they have the same root cause.
  - Similarly, the demonstration of circadian shifts is interesting (lines 409-411), but does not provide causative evidence for the shifts seen in other data.

**Discussion**

- the first sentence of the Discussion draws conclusions about promoter activity when no evidence has been provided to demonstrate this and the second sentence reinforces the tenuous nature of the conclusions drawn, using *“appear to result”* (line 586), which can be taken to emphasise the possible correlation in the absence of evidence of causation
- the logic underpinning the start of the paragraph at line 590 is sound, but the author has not provided the evidence to support it as a conclusion

**Figures and Tables**

- the figure titles are descriptive sentences rather than titles and therefore, it is suggested that they be revised
- clarification in the figure legend of the x-axis values in Figure 1b would be beneficial, as the reader shouldn’t have to refer to the main body of the text to understand the differences in the way in which the data in this figure was binned versus Figures 1c and 1d
  - also, the y-axes are telescoped in b-d, which exaggerates the very small differences between the values, particularly in Figure 1b – a more appropriate scale should be used
- Figure 4 is too small to read and when enlarged, is blurry and therefore, poorly illegible
  - also, “asymmetrical” (“Assymm.”) should be defined
- the format of Supporting Table 1 is awkward: why is the Description column so narrow but the Note column so wide, when the latter generally has little information in it, aside from the URL for GSE59923/etc.? The author is encouraged to reformat this table.
- the table legend for Supporting Table 2 has elements missing
  - CMC should be defined
  - also, the nomenclature (i.e. *“5 d”*, etc.) should be defined

References

- the title of a journal article should be in sentence case, not title case

**Write Style**

- journal guidelines stipulate inclusion of a short title, but this is not present
- abbreviations should be defined before use (e.g. *“…GxE interactions…”* (line 42)*; “…nt…”* (line 146); *“…(NCBI)…”* (line 706); *“…within GEO…”* (line 710))
  - also, it would be beneficial to more formally define “CDS” (lines 148-149) as “coding DNA sequence” and the suggested format would be:
    - *“…protein-coding sequence (coding DNA sequence or CDS) …”*
- there are issues with punctuation, grammar, word choice, phrasing, capitalisation that would have benefited from proofreading, including but not limited to the following:
  - when *“which”* starts a new clause, it must be preceded by a comma (e.g. line 22); in other instances, *“which”* should be replaced with *“that”* (e.g. line 71)
  - *“…for the control and in livers from rats treated…”* (line 87) is grammatically unsound and should be rephrased
  - N-nitrosodimethylamine and glipizide are not capitalised in line 88 but are capitalised in the Figure 1 legend (line 98)
  - the common name (active ingredient) of drugs and compounds is not capitalised (e.g. N-nitrosodimethylamine, glipizide (line 98)); brand/trade names for drugs are capitalised (e.g. Glucotrol, which is one of the brand names for glipizide)
  - it is not necessary to capitalise *“untranslated region”* (line 147)
  - *“moist”* should be *“most”* in line 249
  - do not start a sentence with an Arabic numeral (e.g. line 704)
  - *“e.g.”* is an abbreviation and is the equivalent of writing *“approx.”* in the middle of sentence (e.g. line 708) – either replace it with *“for example”* or put it and the information associated with it in parentheses
  - commas should appear on either side of *“cgr(X)”* (line 721)
  - one sentence, regardless of its length, is not a paragraph
  - casual/colloquial use of “where” (e.g. line 412)
  - why is seemingly unnecessary capitalisation used between lines 478-494?
