# Supplementary material for: A mechanism of global gene expression regulation is disrupted by multiple disease states and drug treatments
Source: PLoS One. 2025 May 8;20(5):e0317071. doi: 10.1371/journal.pone.0317071 (PMC12061403; doi:10.1371/journal.pone.0317071)
Supplement: Attachment — Submitted filename: Response to Reviewers.docx [file pone.0317071.s007.docx]

**PONE-D-24-58748 author response to reviewer comments**

The author would like to express his sincere thanks to the four reviewers for their extensive, thoughtful and helpful comments. Below, the author has set out how each of their points has been addressed, individually or collectively, in the revised manuscript. All line numbers relate to the ‘*track changes, show all changes*’ version.

1) Firstly, apologies to all reviewers for the poor image quality in the original submission, which, I imagine, made the reviewing process much harder than it needed to be. All problem figures have been re-scaled. *The versions visible in the assembled PDF are still not of suitable resolution, but by clicking on the links in the top right of the page the high-resolution versions should download for review.*
2) ‘Data not shown’ has been removed from the MS text (line 477) and, instead, a new **supplemental figure 2** has been created.
3) R#2: The main title and section titles have been substantially reworked and simplified.
4) R#4: The abstract has been substantially reworked to remove reference to Lesch-Nyhan Syndrome (Line 33) which the author agrees was perhaps over-emphasised. Related, R#4: ‘it is recommended that the author reconsider the order in which the data is presented, as this would strengthen the correlations being drawn between the data from the HPRT knockdown cells and certain drugs with the findings in Figures 1-3’. I believe this comment has been addressed by the de-emphasising of Lesch-Nyhan/HPRT in the text and re-wording.
5) R#4: ‘what is meant by *“…simple numerical outputs”*? In the context of an Abstract, this comes across as a bit vague and uninformative’. **RESPONSE:** The abstract text has been changed to provide more explicit detail of the gradient calculation (Line 23).
6) R#4: The reviewer sought clarification of the concepts of supply of nucleobases/amino acids and how this impacts global expression through mRNA or protein sequence composition. The Introduction section now has additional text starting on Line 67 and 83 which hopefully clarifies the logic for the study – which is based on mRNA composition analysis rather than direct nucleobase quantification. The issue of lack of direct quantification of nucleobases is also addressed in further points below.
7) R#3: ‘the authors need to include any potential biases in the dataset selection or analysis methods’, ‘Expand on the mechanistic insights provided by the study, particularly how nucleobase supply and mRNA composition interact at a molecular level.’, and

‘The author needs to expand the conclusion’. R#4:’ it would be helpful for the author to explain how the frequency of individual bases within the transcripts is transformed to demonstrate unequivocally that the promoters do not contribute to altered mRNA levels’. ‘drugs that are alkylating agents will change the availability of certain transcripts due to the DNA damage caused, but how does this correlate with the availability of nucleobases themselves?’,’ Figure 4, which should provide evidence in this regard, is incredibly difficult to read but fluorouracil, for example, had an asymmetrical effect, the meaning of which is left unexplained, and would be expected to skew the transcript profile’. **RESPONSE:** These comments have been grouped together as they all focus on measurement and interpretation of nucleobase supply. They have prompted a much more extensive Discussion section, starting on Line 682, that examines potential confounders, mechanistic explanations, and the issues of very defined drug actions that do not appear to affect nucleobases. Additionally, point 10, below, details further support for the nucleobase supply hypothesis.

8) R#3: ‘Did the author do data scaling? It would be good to normalize or standardize the gene expression data to ensure comparable scales across conditions or genes. For example, z-score transformation can be useful for ensuring comparability. A principal component analysis could also reduce the dimensionality of the data while preserving variance, allowing you to visualize patterns in gene expression across conditions. A PCA plot can summarize the main sources of variation and how different samples or conditions cluster. Another confirmatory tool would be the use of statistical tests such as ANOVA, t-tests, or non-parametric tests (e.g., Kruskal-Wallis) to assess the significance of observed differences across conditions. Present p-values or confidence intervals alongside radar plots to support the interpretations.’.R#3: ‘The author needs to describe their methods, and the design better. The nucleotide composition of any given transcripts are affected by many factors including alternative splicing. It is not clear how the author define it. It is also impossible to estimate the nucleotide composition by computing the GC/AT content in transcriptome data.’ R#4: ‘what constitutes an “outlier” is unclear’. **RESPONSE:** I have addressed these comments together because they largely relate to Methods. Over 100,000 human transcripts were studied derived from transcription of the ~20K protein-coding genes. Thus, multiple splicing is taken into account when calculating compositional information. Equally importantly, most microarray chips used in the generation of the data have the capability to distinguish a proportion of the splice variation in the transcriptome. Thus, the potential action of alternative splicing on the analysis is already accounted for, as much as the data allow. Where mRNA-encoded protein correlations were made, the average mRNA nucleobase composition of all gene X spliceforms was used to compare to protein X amino acid composition. In terms of scaling, the use of gradients or control-corrected gradients is stated in the paper as a means to generate outputs that permit some limited comparison between studies. I did attempt to apply some form of scaling between the 13 studies examining drug effects on global expression profile. This was not successful, and I explain that drug potency on global expression seems to be very tissue-specific (Line 368 and onwards), further convincing me that quantitative cross-study/cross-tissue comparisons are not likely to be possible or biologically useful. Thus, nothing other than direction of effect is shown when outlier drugs are collated in Figure 4. The Supplemental Data 2 for this section does allow others to examine absolute drug potency within studies. On the topic of ‘outlier’ definition, this is visible within Supplemental Data 2, and is already formally defined in the Methods (enhancements made to text description), and the legends to Figs. 3 and 4 (again, text added). The statistical analysis requested is present in Figure 6 in the form of 95% confidence interval limits, and the labelled instances where two-tailed T-tests indicated statistically significant profile differences between conditions. Again, I must apologise for the resolution of the images obscuring this valuable information. Overall, I have responded to these comments by adding greater detail to the Methods section of the paper.
9) R#4: ‘were the bins independent of the tissue type is the early studies?’ and ‘there appears to be a contradiction between the information in the Methods section, lines 716-717, which states that transcripts, “…were divided into 25 equal-sized bins from the lowest to highest frequency…” and the comment in line 86 that there were “…25 equal-sized bins each containing hundreds of randomly allocated transcripts”’. **RESPONSE:** Bins only relate to transcript composition and not to tissue origin. They are used to visually simplify the complexity of global expression and to provide a means to calculate the ‘gradient’ of the global profile. The actual analyses of nucleobase effects on expression are always conducted on bins of compositionally sorted transcripts (lowest frequency of base X in bin 1 all the way to highest frequency of base X in bin 25). However, when this concept is first introduced on Line 96, a ‘sham’ analysis (Fig.1b) without ranking was applied such that when compositional ranking was actually introduced (Fig.1c and Fig.1d), the impact on the gradient becomes immediately apparent. I have re-phrased the analytical description in this section to remove any confusion and to explicitly label and highlight the ‘sham’ comparison.
10) R#4: ‘since there aren’t any direct measures of free nucleobase quantities or promotor activity in this section, it would be helpful for the author to explain how the frequency of individual bases within the transcripts is transformed to demonstrate unequivocally that the promoters do not contribute to altered mRNA levels’, ‘Repeatedly across Figures 1-3, the author draws conclusions about the nucleobase supply, correlating it with nucleobase composition in the absence of evidence that the supply is perturbed. I have chosen not to enumerate all of the statements of concern, as this would have run to many pages, but certain key issues are identified below.’. ‘the strongest data in this study comes from the HPRT knockdown cells (lines 373-377). That said, correlation does not equal causation, and therefore, the fact that the C+A- global transcriptome change seen in these cells resembles that seen in other data does not mean that they have the same root cause.’ And ‘the first sentence of the Discussion draws conclusions about promoter activity when no evidence has been provided to demonstrate this and the second sentence reinforces the tenuous nature of the conclusions drawn, using “appear to result” (line 586), which can be taken to emphasise the possible correlation in the absence of evidence of causation’ **RESPONSE:** The reviewer indicates a number of statements and claims throughout the manuscript which perhaps rely too heavily on the initial assumptions of a nucleobase supply model. I have collated many of these comments in this response point. In addition to new text in the Introduction that acknowledges the lack of direct nucleobase quantification (Line 67 and, separately, 83), I have added two new analyses to accompany the data in Figure 3 (from Line 290, and **supplemental Figure 1**). They cannot replace direct quantification of nucleobases as a gold standard, but I think they convincingly support the hypothesised mechanism and therefore justify claims made elsewhere. As a basis for the first analysis, I claim (and this is implicit in the model) that there will be an equilibrium between the free nucleobase pool and the pool of nucleobases incorporated into the total cellular mRNA. This allows the inference of nucleobase supply change after drug treatment by determining if the relative abundance of each of the nucleobases locked up in cellular transcripts has changed (an actual measure that is possible to make from the expression data). This is shown to be the case. Secondly, it is determined that two very different drugs (structure/pharmacological action) can be selected - but with similar gradient values and direction - and shown to have almost identical influences on global expression change (gene identity and expression level correlation). I believe these two new observations cannot be reconciled with a promoter-based explanation and, instead, support a ‘universal’ influence on gene expression based on changing nucleobase supply coupled with unchanging mRNA composition. I thank the reviewer for demanding better supportive evidence for the theoretical claims.
11) R#4: ‘it is unclear how coding DNA sequences that result in proteins rich in essential amino acids provides any insight into nucleobase availability (lines183-187) or why this study was needed to illustrate a skew in the nucleobases required for the different amino acids. Simply counting the different bases in the genetic code across the types of amino acids would have demonstrated this without the need for experimentation and therefore, the comment that it was “unexpected” seems unusual. I have obviously missed the point and therefore, would find it helpful for the author to provide additional information to explain their meaning.’ And ‘ untranslated regions are known to regulate expression (e.g. 5’UTR regulates ribosome recruitment, translation efficiency, etc.) and therefore, protein availability, so how does the nucleobase composition of 5’UTR or 3’UTR provide insights into nucleobase availability (lines 202-205)?’. **RESPONSE:** The ‘prequel’ paper to this one (Thompson & Pickard, 2024) identified two of the three nutritionally classified amino acid groupings (essential, conditionally essential amino acids) as constraints on protein expression, much as A+U, and C+G nucleobases are identified as constraints on mRNA expression in this manuscript. I wished to test whether there was any formal relationship between these two very distinct forms of building-block supply constraint on expression level. Remarkably, A+U enriched (=A+U supply susceptible) transcripts preferentially encode proteins enriched in essential amino acids, whereas C+G enriched transcripts encode conditionally essential amino acid enriched proteins. That is unexpected because it is a bit like arbitrarily dividing a room full of many people into two groups and finding one group owns cats and the other dogs. It seems incredibly unlikely. However, this nucleobase-amino acid alignment may offer a clue to its origin/purpose: any metabolic shortfall in A+U has ‘reach-through’ to reduce the synthesis of essential amino rich proteins. I proposed in the text that the risks of starvation conditions (in which there is no essential amino acid intake) include causing significant cellular stress, as ribosomes try but fail to make proteins with many essential amino acids in them. This stress might be mitigated, in part, by those same starvation conditions affecting nucleobase metabolism, reducing A+U availability, and therefore the reducing transcription of mRNAs likely to code for those ‘dangerous’ essential amino acid-rich proteins. In fact, I went one stage further by claiming that, while the mutational loss of biochemical machinery for synthesising essential amino acids in higher organisms might be of energetic benefit, it could only have happened in the context of the safeguarding offered by global mRNA expression control. One more aspect is discussed (and raised as an issue by R#4): the observation that the 5’UTR or 3’UTR non-coding sequences of a transcript also influence amino acid type in their encoded proteins. While it’s intuitive that coding regions offer reach-through to protein in the global expression model, it seems to be the case that it has been selectively beneficial for UTR sequences to evolve a shared similarity in nucleobase composition to their adjoining ORFs because this would increase the sensitivity of the entire transcript to a nucleobase supply-mediated form of expression regulation. I believe this speculation is warranted as it provides a testable hypothesis for the origin and original purpose of global expression control and the emergence of the animal kingdom (and also suggests yet another function for UTRs). It also links diet (=environment) with the disease pathologies described (=genes), which I believe is a useful route into the underdeveloped gene x environment (GxE) model of disease susceptibility. Starting at Line 191 and Line 727 additional text and a relevant, supportive reference has been added.
12) R#4: regarding the number of replicate treatments (line 241), what does “approximately twelve replicate treatments” mean? **RESPONSE:** The legend has been altered for clarity: “Data points represent the average of the biological replicates of expression quantification for each drug, with replicates typically numbering twelve.”
13) R#4: limitations of this work (lines 221-223) belong in the Discussion, not the Results. **RESPONSE:** text moved to Discussion
14) R#4: ‘the figure titles are descriptive sentences rather than titles and therefore, it is suggested that they be revised’. **RESPONSE:** legend titles have a been shortened and altered to indicate both method and principal outcome.
15) R#4: ‘“asymmetrical” (“Assymm.”) should be defined’ **RESPONSE:** Text on Line 239 has been expanded
16) R#4: ‘clarification in the figure legend of the x-axis values in Figure 1b would be beneficial, as the reader shouldn’t have to refer to the main body of the text to understand the differences in the way in which the data in this figure was binned versus Figures 1c and 1d. Also, the y-axes are telescoped in b-d, which exaggerates the very small differences between the values, particularly in Figure 1b – a more appropriate scale should be used.’ **RESPONSE:** The figure has been revised accordingly to clarify X-axis labels. However, the Y-axis scale has been maintained. The lines drawn between 1a and 1b openly signpost this compression of the Y-axis – a property of averaging hundreds of expression values. Similarly, the small scale of the deviations (statistical significance confirmed throughout the manuscript, and error bars used here) is acknowledged in the text and proposed as being constrained in magnitude by toxicity – the most extreme outlier drugs are often toxins or have been withdrawn from therapeutic use due to adverse effects.
17) ‘Unfortunately, in places, the text reads as if the data is being constrained to justify the hypothesis, rather than the conclusions arising directly from the data. Additionally, some of the conclusions seem to stretch a bit too far, particularly considering the absence of direct measures of nucleobase availability and promotor activity.’ **RESPONSE:** It is also believed that the two new sections mentioned in point 10, above, provide a more solid foundation for any claims made that involve nucleobase supply. However, the author has made changes to the Discussion section to improve clarity and reduce the reach of some claims, although a certain level of speculation is perhaps useful to frame the ‘how and why’ of a new concept, particularly when that speculation is experimentally testable in the future.
18) R#4: ‘the format of Supporting Table 1 is awkward’ **RESPONSE:** Table column dimensions have been adjusted.
19) R#4: ‘the table legend for Supporting Table 2 has elements missing’ RESPONSE: The legend has been expanded and the Table has been modified by the removal of non-essential information such as the treatment duration (all of which can be obtained from the original datasets and publications).
20) R#4: ‘the title of a journal article should be in sentence case, not title case’ **RESPONSE:** all references are formatted via Endnote in PloS style.
21) R#4: ‘journal guidelines stipulate inclusion of a short title, but this is not present’ **RESPONSE:** This was included in the submission and is on the PDF front page: ‘Regulation of gene expression at a global scale’
22) R#4: The numerous abbreviation, typographical, consistency and punctuation errors have been corrected throughout the MS.
